# The role of cancer-associated mesothelial cells in the progression and therapy of ovarian cancer

**DOI:** 10.3389/fimmu.2022.1013506

**Published:** 2022-10-04

**Authors:** Aiping Zheng, Yuhao Wei, Yunuo Zhao, Tao Zhang, Xuelei Ma

**Affiliations:** ^1^ Division of Biotherapy, Cancer Center, West China Hospital, Cancer Center, Sichuan University, Chengdu, China; ^2^ Head & Neck Oncology Ward, Cancer Center, West China Hospital, Cancer Center, Sichuan University, Chengdu, China; ^3^ West China School of Medicine, West China Hospital, Sichuan University, Chengdu, China

**Keywords:** ovarian cancer, cancer-associated mesothelial cells, tumor progression, chemoresistance, tumor therapy

## Abstract

Ovarian cancer is currently one of the most common malignant tumors in females with poor survival rates around the world, killing about 200,000 women each year. Although great progress has been made in treatment, most patients receiving first-line therapy experience tumor recurrence. The tumor microenvironment plays an important role in regulating the progression and prognosis of ovarian cancer. Cancer-associated mesothelial cells are the main cell population in the tumor microenvironment, which affect the progression, prognosis and chemical resistance of ovarian cancer. Cancer-associated mesothelial cells can also interact with other microenvironmental components, such as exosomes, macrophages, and adipocytes. Some studies have developed drugs targeting cancer-associated mesothelial cells in ovarian cancer to evaluate the therapeutic efficiency. In this review we highlighted the key role of cancer-associated mesothelial cells in the progression and prognosis of ovarian cancer. We also described the progress of cancer-associated mesothelial cells targeted therapy for ovarian cancer. Continued insight into the role of cancer-associated mesothelial cells in ovarian cancer will potentially contribute to the development of new and effective therapeutic regiments.

## Introduction

Ovarian cancer (OC) is a common malignant cancer among women in the world and has a poor prognosis (1). According to the latest world health organization data, 313959 patients are diagnosed with OC and 207252 die from it worldwide annually (2). Current frontline treatment for OC consists of initial debulking surgery and subsequent consolidation chemotherapy. 70-80% of patients experience recurrence after standard frontline treatment, making the five-year survival reach about 45% (3). The high mortality and recurrence rates in OC patients are mainly due to chemotherapy resistance and widespread abdominal metastasis. Recently, immune therapeutics have been introduced to the ovarian cancer treatment landscape, including but not limited to immune checkpoint inhibition (ICI), tumor antigen vaccines and engineered immune cells (4). However, some clinical trials testing ICI in OC have not delivered positive results (5). More effective treatment is urgently in need.

Tumor microenvironment (TME) plays a key role in tumor progression and response to standard chemotherapy. A ton of basic and preclinical studies suggests that co-treatment targeting TME improves therapeutic effect (6). Cancer-associated mesothelial cells (CAMs) are a major part of the OC microenvironment, contributing to cancer progression and chemoresistance. This review summarizes how CAMs obviously influence the progression and prognosis of OC and reviews several targeted therapies for CAMs.

## The effects of CAMs in OC

The peritoneum, the omentum and the serous membranes of the small intestine and large intestine are covered by monolayer mesothelial cells. These cells are the primary barrier to prevent the dissemination of OC cells. Like other epithelial cell layers in the body, such as cervical epithelium, the mesothelial cells act as a protective barrier to protect the underlying tissue from OC cells and limit access to the retroperitoneum. A previous study found that primary human mesothelial cells inhibited the initial adhesion and invasion of at least two OC cell lines and three different early passage human OC cell cultures (7). However, in patients with OC, cancer cells can secrete a series of cancer-promoting factors to induce the mesothelial-mesenchymal transition (MMT) of normal mesothelial cells (8). We defined mesothelial cells differentiated by OC cells stimulation as cancer-associated mesothelial cells (CAMs). Compared with mesothelial cells, CAMs undergo obvious morphological changes, and the polarity of cytoskeleton becomes disordered. CAMs also showed significant epithelial-mesenchymal transition (EMT) characteristics, such as the increase of fibronectin, α-SMA and vimentin and reduction of E-cadherin. CAMs no longer have a protective effect, but secrete chemokines to promote the peritoneal metastasis and chemoresistance of ovarian cancer cells.

### CAMs and peritoneal metastasis in OC

During the process of peritoneal metastasis of OC, CAMs promote the adhesion and invasion of OC cells to the peritoneum through regulating the expression of multiple chemokines. A recent study found that the expression of intelectin-1 (ITLN1) in CAMs and serum ITLN1 levels in OC patients were significantly lower than those in healthy women. ITLN1 fused with lactotransferrin (LTF) and dampened the binding of LTF to its receptor on the surface of OC cells, low-intensity lipoprotein-receptor-related protein 1(LRP1). ITLN1 attached to LRP1 and transcriptionally increased the expression of MMP1, which contributed to the invasion and metastasis of cancer cells. Simultaneously, ITLN1 inhibited the invasion ability of OC cells by suppressing LTF-induced calcium mobilization and stress fiber formation. In addition, ITLN1 increased recombinant glucose transporter-4 (GLUT4) production in adipocytes, which contributed to increased glucose uptake by adiposes and decreased glucose uptake by tumor cells, thereby the proliferation of OC cells was inhibited. *In vivo* experiments, treatment with recombinant ITLN1 inhibited OC growth (9). In ovarian cancer, hypoxic microenvironment induced CAMs and cancer cells to stabilize HIF-1 and HIF-2. HIF signaling upregulated collagen prolyl 4-hydroxylases (P4HA1, P4HA2 and P4HA3), lysyl hydroxylases (PLOD1 and PLOD2) and lysyl oxidase (LOX) to facilitate the crosslinking and deposition of extracellular collagen type I in mesothelial cells, finally contributed to OC cells metastasis (10). CAMs can also secret several cytokines to promote the metastasis of OC. CAMs were reported to increase the secretion of IL-8 (11) and CCL2 (12). IL-8 secreted by CAMs induced the overexpression of pyruvate dehydrogenase kinase-1 (PDK1) in OC cells *via* CXCR1. PDK1 upregulated the expression of α5 and β1 integrin to enhance the adhesion to fibronectin and mesothelial cells. PDK1 also activated JNK signaling to induce IL-8 production in OC cells (11). In addition, IL-8 bound to CXCR1 and CXCR2 on endothelial cells situated on subperitoneal tissue to promote tumor neovascularization (13). CCL2 facilitated the trans-mesothelial migration and invasion of OC cells *via* activating p38-MAPK pathway through CCR2 (12). Pericellular hyaluronic acid (HA) secreted by CAMs can bind to CD44v3-Vav2 complex on OC cells to activate RhoGTPase (Rac1) pathway signaling, in turn, promoted the activation of cytoskeleton, finally facilitating cancer cells invasion. Simultaneously, HA bound to CD44v3- p185^HER2^ complex to promote p185^HER2^ tyrosine kinase (TK) activation, and then the adaptor molecule Grb2 was recruited. Grb2 not only activated Ras pathway signaling to regulate cancer cells growth, but also interacted with Vav2 to activate Rac1 pathway signaling (14). High levels of Wnt5a deriving from CAMs in ascites fluid boosted the metastasis of OC cells *via* activating its downstream effector Src family kinase Fgr (15). A previous study found that CAMs generated lysophosphatidic acid (LPA) *via* cytosolic phospholipase A2 (cPLA2) and calcium-independent phospholipase A2 (iPLA2) activity to activate extracellular signal-regulated kinase (ERK) and Akt pathway in OC cells, in turn, boosted OC cells to adhere to collagen I, finally promoted the metastasis of OC (16).

The intricate crosstalk between CAMs and cancer cells facilitates the metastasis of OC. Transforming growth factor-β (TGF-β) derived from OC cells induced the phenotypic changes of mesothelial cells to CAMs (17). CAMs increased the secretion of vascular endothelial growth factor (VEGF) in a TGF-β-dependent manner. VEGF secreted by CAMs acted on endothelial cells situated in subperitoneal space and boosted their migratory potential and tube formation ability, thereby promoting tumor neovascularization (18). TGF-β also activated RAC1/SMAD3 pathway *via* TGF-βRII to induce CAMs to upregulate fibronectin expression. Fibronectin in extracellular matrix binds to α5 and β1 integrin on OC cells to support the metastasis (19). Moreover, extrinsic TGF-β derived from OC cells induced the extra secretion of TGF-β from CAMs, leading to a cumulative effect of TGF-β (20). A previous study showed that OC cells secreted hepatocyte growth factor (HGF) to induce MCs to differentiate into CAMs (21). Hepatocyte growth factor (HGF) derived from OC cells also promoted the premature senescence of normal mesothelial cells by inducing mitochondrial oxidative stress *via* activating several signaling pathways including p38-MAPK, AKT and NF-κB (22). Senescent mesothelial cells upregulated the expression of fibronectin (FN) (23) and downregulated the expression of junctional proteins, such as connexin 43, E-cadherin, occludin and desmoglein, leading to destruction of the integrity of the peritoneal mesothelium and makes it easier for the invasion of ovarian cancer (24). Senescent mesothelial cells also secreted angiogenic agents such as CXCL1, CXCL8 and VEGF to stimulate subperitoneal tumor neovascularization (25). In addition, OC cells overexpressed plasminogen activator inhibitor-1 (PAI-1) and transcription factor DLX4 to induce the expression of IL-8/CXCL5 and IL-1β/CD44 *via* activating NF-κB signaling, further enhancing tumor-mesothelial cell interactions and facilitating the metastasis (26, 27).

**Figure 1:**
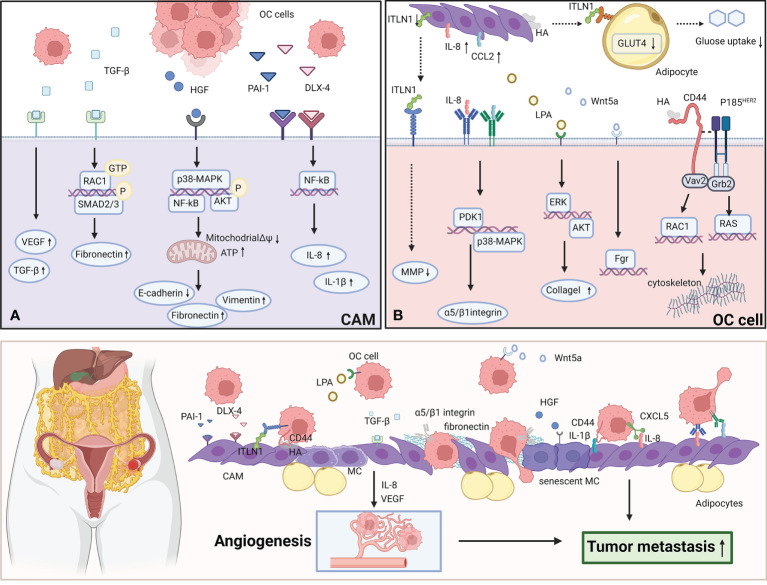
CAMs interact with OC cells to promote the metastasis. **(A)** OC cells secret TGF-β, HGF, PAI-1, DLX-4 to effect CAMs *via* various signaling pathways. TGF-β activates RAC1/SMAD3 pathway *via* TGF-βRII to induce CAMs to upregulate fibronectin expression. HGF promotes the premature senescence of normal mesothelial cells by inducing mitochondrial oxidative stress *via* activating several signaling pathways including p38-MAPK, AKT and NF-κB. PAI-1 and DLX4 induce the expression of IL-8/CXCL5 and IL-1β/CD44 *via* activating NF-κB signaling. **(B)** CAMs overexpress ITLN1, IL-8, CCL2, LPA, Wnt5a and HA to effect OC cells by activating several signaling pathways. IL-8 induces the overexpression of PDK1 in OC cells *via* CXCR1.PDK1 upregulates the expression of α5 and β1 integrin to enhance the adhesion to fibronectin and mesothelial cells. CCL2 facilitates the trans-mesothelial migration and invasion of OC cells *via* activating p38-MAPK pathway through CCR2. Wnt5a boosts the metastasis of OC cells *via* activating its downstream effector Src family kinase Fgr. LPA activates ERK and Akt pathway to boost OC cells to adhere to collagen I. HA can bind to CD44v3-Vav2 complex on OC cells to activate Rac1 and Ras pathway signaling. The figure was created with BioRender.com.

A schematic illustration of the interaction between CAMs and OC cells to promote metastasis is shown in **Figure 1**.

### CAMs and chemoresistance in OC

Chemoresistance is a primary drawback in the treatment of OC. Multiple studies have demonstrated that HA-CD44 interaction facilitated chemoresistance in various cancers *via* several signaling, such as breast cancer and multiple myeloma (28, 29). In OC, the binding of HA to CD44-Nanog complex activated the expression of Nanog-special target genes Rex1 and Sox2. Nanog activation was determined to be closely related to maintaining the stem cell properties of cancer cells. Some activated Nanog interacted with STAT3 to upregulate the expression of multidrug resistance-1 (MDR1) gene, which contributed to the chemoresistance of cancer cells. In addition, HA facilitated the interaction of ankyrin-MDR1 (P-gp) with CD44 and the complex led to chemotherapeutic drugs efflux in OC cells (30). Similarly, another study found that HA induced the expression of membrane ATP binding cassette (ABC) transporter proteins in OC cells to increase chemo resistance (31).

**Figure 2:**
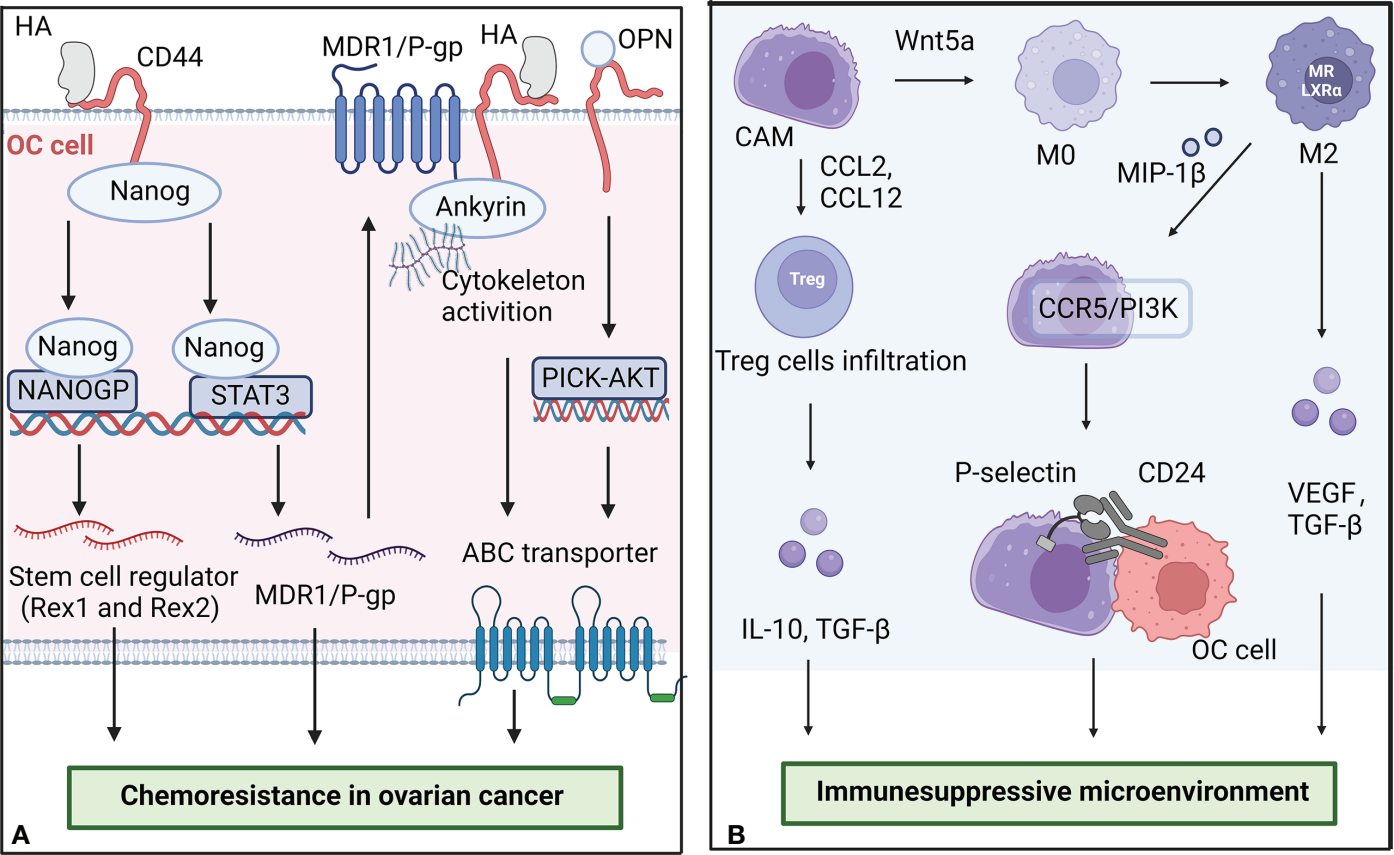
The role of CAMs in chemoresistance and the formation of immunesuppressive microenvironment. **(A)** CAMs secret HA and OPN to promote the chemoresistance of OC cells. The binding of HA to CD44-Nanog complex activated the expression of Nanog-special target genes Rex1 and Sox2. Some activated Nanog interacted with STAT3 to upregulate the expression of multidrug resistance-1 (MDR1) gene. OPN activated HA/CD44/PI3K-AKT signaling pathway to promote the expression of ABC transporter proteins. **(B)** CAMs interact with other cells in the microenvironment to promote the formation of immunesuppressive microenvironment in OC. CAMs can secrete Wnt5a to regulate macrophage polarization and increase T regulatory cell infiltration. M2 macrophages promote the adhesion of CAMs and OC cells by overexpressing MIP-1β. The figure was created with BioRender.com.

Recently, a study found that CAMs can secret osteopontin (OPN) to media chemoresistance and stemness in OC. OC cells induced CAMs to upregulate the expression and secretion of OPN in a TGF-β dependent manner. OPN activated HA/CD44/PI3K-AKT signaling pathway to promote the expression of ABC transporter proteins and regulate BCL-2/BAX ratio, finally contributed to boosting chemoresistance (32). Another mechanistic study indicated that the overexpression of FN in CAMs also reduced platinum-sensitivity in OC cells by activating Akt signaling pathway (33). Moreover, the OC spheroids display enhanced resistance to anti-cancer drugs compared to monolayers, while CAMs promoted spheroid formation by OC cells and induced their motility (34, 35). Chemoresistance OC cells showed a higher ability to adhere and grow on mesothelium, which enhances the dissemination and invasion of cancer cells. A schematic illustration of CAMs promoting chemoresistance in OC is shown in **Figure 2A**.

### CAMs and prognosis of OC

Peritoneal metastasis and chemoresistance dramatically influence the prognosis of OC. Secretion of CAMs such as OPN and ITLN1 have been demonstrated to predict overall survival rates in mice (9, 32). A recent scRNA-seq study, which analyzed 18,403 cells gathered from seven untreated patients with high-grade serous tubo-ovarian cancer, identified 6 cellular phenotypes associated with prognosis (36). It was found that concentration of CAMs was correlated with poor outcome. A prospective observational cohort study found that the expression of vascular cell adhesion molecule-1 (VCAM-1) on the CAMs negatively correlated with progression-free and overall survival in OC (37). Moreover, patients with consistently high VCAM-1 expression were more likely to develop platinum resistance than patients expressing low VCAM-1.

## CAMs and microenvironment in OC

The highly inhibitory immune microenvironment is considered to be one of the dominant reasons for tumor progression and treatment failure in OC patients. As a key part of the tumor microenvironment, CAMs interact with other cells in the microenvironment to regulate the progression of OC. A schematic illustration of CAMs interacting with other cells in the microenvironment in OC is shown in **Figure 2B**.

### CAMs and exosomes

Exosomes are 30-100nm membrane vesicles of endocytosis origin, mediating cell-cell communication and antigen presentation *via* transferring proteins, mRNAs, and microRNAs (38). Recent reports displayed that tumor-derived extracellular exosomes played an important role in communication between CAMs and OC cells to induce immunosuppression, thereby promoting the direct adhesion and invasion of cancer cells to CAMs. For example, OC-derived exosomes carrying CD44 reprogramed mesothelial cells to a more EMT phenotype, which facilitated cancer adhesion and invasion (39). Similarly, *via* co-culturing exosomal annexin A2 (ANXA2) derived from OC cells with human peritoneal mesothelial cells, researchers found that ANXA2 activated PI3K/AKT/mTOR pathway to promote MMT and the degradation of the extracellular matrix of mesothelial cells, finally facilitating establishing pre-metastasis microenvironment of OC (40). In addition, some researchers proposed that OC-derived extracellular vesicles containing MMP1 mRNA induced the apoptosis of mesothelial cells, which exposed the underlying tissue and facilitated peritoneum colonization (41).

### CAMs and macrophages

CAMs can secrete Wnt5a to regulate macrophage polarization. High levels of Wnt5a in ascites fluids activated the Src family kinase Fgr to enhance the immunosuppressive immune landscape of OC and promote peritoneal colonization. The knockout of Wnt5a contributed to an increase in M1 macrophages and a decrease in M2 macrophages in a mouse model of ovarian cancer (15). Generally, M1 macrophages secrete proinflammatory cytokines such as TNF-α, IL-1 and IL-6, which promotes anti-tumor immune response and enhances immune monitoring, while M2 macrophages mediate immunosuppressive response and promote chronic inflammation and tumor invasion mainly by secreting inhibitory cytokines such as TGF-β, VEGF and MMPs. Moreover, Wnt5a expression increased CCL2, CCL12, CXCL10, and CXCL12 production, which correlating with T regulatory cell and tumor-associated macrophage infiltration.

CAMs influence macrophage polarization, while macrophages can also regulate CAMs to promote the adhesion and invasion of ovarian cancer. M2 macrophages can secrete MIP-1β to activate CCR5/PI3K signaling and then increase P-selectin production by CAMs. P-selectin binds to CD24 on the OC cells surface, leading to increased adhesion of cancer cells (42). Anti-P-selectin antibody and small molecular inhibitor were demonstrated to inhibit OC cells adhesion *in vivo* and *in vitro*.

### CAMs and cancer-associated fibroblasts (CAFs)

During peritoneal metastasis, tumor cells can induce the transformation of mesothelial cells into CAFs. A previous study found the presence of CAFs expressing mesothelial markers at the site of tumor implantation in patients with peritoneal dissemination (8). By single-cell RNA sequencing and spectral tracing assays, a recent study demonstrated that antigen-presenting CAFs were derived from mesothelial cells. Further study revealed that IL-1 and TGF-β can induce mesothelial cells to downregulate mesothelial features and acquire fibroblastic features during tumor progression. Antigen-presenting CAFs induced regulatory T cells formation and expansion in an antigen-specific manner, which contributes to tumor immune escape (43).

### CAMs and adipocytes

OC cells are prone to metastasize to the omental fat pad (44). Omental adipocytes release and transport free fatty acids to maintain the high energy requirements of cancer cells (45). In diet-induced obesity mice, the density of microvilli on peritoneal mesothelial cells was significantly increased, which contributes to early cell-cell adhesive events in metastatic colonization in ovarian cancer (46). Moreover, the downregulation of ITLN1 in CAMs inhibited the insulin-dependent glucose uptake in mature adipocytes *via* suppressing the expression of GLUT4, which increased the glucose available to OC cells and promote the tumor growth (9).

## Targeting CAMs in therapy of OC

CAMs interact with OC cells *via* expressing some specific markers, such as MSLN, FN and HA. Some studies have developed antibodies targeting these markers to block the interaction of CAMls with OC cells, thereby inhibiting the progression of OC and improving the prognosis of OC survivors.

### MUC16 - Mesothelin

MUC16 is a glycoprotein that is overexpressed by OC cells. The shedding of MUC16 from the surface of OC cells to circulation is the basis of serum assay CA125 in clinical. Mesothelin (MSLN) is a differentiation antigen mainly expressed on CAMs, OC cells and mesothelioma cells. MUC16–MSLN interaction mediated the attachment and adhesion of OC cells to CAMs. Blocking the MUC16–MSLN interaction can effectively inhibit cancer cell adhesion and invasion.

Initially, antibodies against MUC16 were developed for the immunotherapy of OC, such as murine IgG1 oregovomab (mAb B43.13, OvaRex) (47). However, the treatment with oregovomab monotherapy failed to prolong the survival of patients with advanced OC in a phase III clinical trial (48). The combination therapy with oregovomab plus carboplatin/paclitaxel effectively improved overall and progression-free survival (49). Some clinical trials of oregovomab in combination with other drugs are also underway, such as bevacizumab and niraparib (ClinicalTrials.gov identifier: NCT04938583; NCT05335993). Later, Antibody–drug conjugates (ADCs) targeting the repetitive MUC16 epitopes were developed (50), which were revealed to have higher antitumor activity, such as 3A5-MMAE (monomethyl auristatin E) (51). Constructing chimeric antigen receptor (CAR) targeting to the retained extracellular domain of MUC16 (MUC-CD) has also been demonstrated to exhibit efficient antitumor activity *in vitro* and *in vivo (* 52). Recently, a human bispecific T-cell engaging antibodies (REGN4018) bridging MUC16-expressing cells with CD3 T cells was developed (53). In preclinical studies and toxicology studies, REGN4018 displayed potent antitumor activity and good tolerability. Moreover, the combination of bispecific T-cell engaging antibodies and anti-VEGF enhanced the efficacy (54). Using an oncolytic adenovirus carrying a MUC16- bispecific T-cell engagers (BiTEs) can activate and retarget CTLs to enhance the anti-tumor effect (55). Currently, Phase 1/2 trails are recruiting patients (ClinicalTrials.gov identifier: NCT03564340; NCT04590326).

MORAb-009, a chimeric antibody targeting MSLN, is being investigated in multiple clinical studies. Patients with MORAb-009 treatment exhibited more stable disease and clear increase in serum MUC16, which suggesting MORAb-009 disturbs the MSLN–MUC16 interaction (56). Anti-MSLN antibody-drug conjugate anetumab ravtansine is composed of a human anti-MSLN IgG1 and a maytansine derivative tubulin inhibitor DM4, which shows selective and potent antitumor activity in xenograft tumor models (57). In a phase I multicenter trial, anetumab ravtansine displayed a manageable safety, favorable pharmacokinetics and preliminary antitumor activity in patients with mesothelin-expressing solid tumors (58). Additionally, A randomized phase II Study is underway (ClinicalTrials.gov identifier: NCT03587311). Anti-MSLN CAR-T cells are in progress with some clinical trials (ClinicalTrials.gov identifier: NCT04562298; NCT04503980). In orthotopic mouse models of OC, MSLN-directed CAR T cells provided antitumor immunity and significantly prolonged survival (59). Clinical trials of MUC16-MSLN targeted drugs are described in detail in **Table 1**.

**Table 1:** Summary of clinical trials using MUC16-mesothelin (MSLN) and FN - α5β1 integrin targeted agents.

Target	Agent	Type of clinical trial	Patient population	Enrollment	Status	ClinicalTrials.gov Identifier
HGF	Rilotumumab	Phase II	Patients with recurrent or persistent ovarian cancer	31	Completed	NCT01039207
MUC16	Oregovomab	Phase II	Patients with ovarian cancer (FIGO Stage III or IV)	102	Terminated	NCT00034372
	Oregovomab	Phase III	Patients with ovarian cancer (FIGO Stage III or IV)	354	Terminated	NCT00050375
	Oregovomab	Phase II	Patients with ovarian, fallopian tube, or peritoneal cancer	102	No known	NCT00004064
	Oregovomab	Phase II	Patients with residual disease from stage III or stage IV ovarian epithelial, fallopian tube, or peritoneal cancer following surgery and chemotherapy	400	No known	NCT00003634
	Oregovomab +Carboplatin + Paclitaxel	Phase II	Patients with advanced ovarian cancer	97	Completed	NCT01616303
	Oregovomab +Bevacizumab +Paclitaxel +Carboplatin	Phase I/II	Patients with BRCA wild type platinum sensitive recurrent ovarian cancer	54	Recruiting	NCT04938583
	Oregovomab +Nivolumab	Phase I/II	Patients with epithelial cancer of ovarian, tubal or peritoneal origin	13	Terminated	NCT03100006
	Oregovomab +Poly ICLC	Phase I	Patients with CA125-associated, advanced ovarian cancer (FIGO Stage III/IV)	10	Terminated	NCT03162562
	Oregovomab +Paclitaxel + Carboplatin +Placebo	Phase III	Patients with advanced epithelial ovarian cancer following optimal debulking surgery	602	Recruiting	NCT04498117
	Oregovomab + Nivolumab + Chemotherapy	Phase I/II	Patients with epithelial cancer of ovarian, tubal or peritoneal origin	31	Recruiting	NCT04620954
	Oregovomab +PLD	Phase II	patients with PARP inhibitor-resistant ovarian cancer	28	Recruiting	NCT05407584
	Oregovomab +Niraparib	Phase II	Patients with platinum sensitive recurrent ovarian cancer.	10	Recruiting	NCT05335993
	DMUC5754A	Phase I	Patients with platinum-resistant ovarian cancer or unresectable pancreatic cancer	77	Completed	NCT01335958
	REGN4018	Phase I/II	Patients with recurrent ovarian cancer	554	Recruiting	NCT03564340
	REGN4018	Phase I/II	Patients with recurrent ovarian cancer	326	Recruiting	NCT04590326
mesothelin (MSLN)	MORAb-009	Phase I	Patients with mesothelin-positive cancers: ovarian, pancreatic, mesothelioma, non-small cell lung cancer.	24	Completed	NCT00325494
	MORAb-009	Phase I	Patients with mesothelin-positive cancers: ovarian, pancreatic, mesothelioma, non-small cell lung cancer.	6	Completed	NCT01521325
	MORAb-009	Early phase I	Patients with mesothelin-positive cancers: ovarian, pancreatic, mesothelioma, non-small cell lung cancer.	7	Terminated	NCT01413451
	Anetumab Ravtansine +Pegylated Liposomal Doxorubicin	Phase I	Patients with ovarian cancer	65	Completed	NCT 02751918
	Anetumab Ravtansine + Bevacizumab + Paclitaxe	Phase I	Patients with refractory ovarian, fallopian tube, or primary peritoneal cancer	96	Active, not recruiting	NCT03587311
	LCAR-M23 (CAR-T cell)	Phase I	Patients with relapsed and refractory epithelial ovarian cancer	34	No known	NCT04562298
	αPD1-MSLN-CAR T cells	Early phase I	Patients with MSLN-positive advanced solid tumors: ovarian cancer, cholangiocarcinoma, colorectal cancer	10	Recruiting	NCT04503980
α5β1 integrin	Volociximab +Liposomal Doxorubicin	Phase I/II	Patients with advanced epithelial ovarian cancer or primary peritoneal cancer relapsed after prior therapy with Plat/Taxane-based chemo	138	Completed	NCT00635193
	Volociximab	Phase II	Patients with platinum-resistant, advanced epithelial ovarian or primary peritoneal cancer	16	Terminated	NCT00516841

### FN - α5β1 integrin

FN secreted by CAMs is one of the most abundant extra-cellular matrix proteins in the peritoneal microenvironment. OC cells can adhere to FN *via* α5β1 integrin and directly induce phosphorylation of focal adhesion kinase (FAK), further leading to activation of mitogenic pathways supporting tumor growth (60). Blocking antibodies against α5β1 integrin effectively inhibited OC cells adhesion to mesothelial cells. Volociximab is a high-affinity, chimeric antibody directed against human α5β1 integrin. However, in a phase II, single-arm study, volociximab treatment failed to achieve sufficient clinical activity in patients with recurrent, platinum-resistant ovarian cancer (61). The disappointed result may be related to the use of a single agent intervention in recurrent and advanced OC patients. Combination therapy with volociximab in low-volume residual disease after cytoreductive surgery or as maintenance therapy to prevent recurrence of ovarian cancer may be effective. Moreover, a previous study reported that resveratrol decreased cellular α5β1 integrin level to inhibit ovarian cancer cell adhesion to CAMs *in vitro (* 62).

Tissue transglutaminase (TG2) is a transpeptidase that promotes the formation of FN - α5β1 integrin complexes by interacting with FN. A function-inhibiting antibody against the TG2 FN-binding domain suppressed complexes formation and blocked the proliferation of cancer stem cells (63). Compound ITP-79 inhibited the binding of TG2 peptide to the 42-KDA FN fragment in a dose-dependent manner, thereby disrupting FN - α5β1 integrin complexes and blocking the adhesion of cancer cells to mesothelial cells (64). FN - α5β1 integrin complexes targeting strategies need to be further optimized and tested for safety, tolerability and efficacy in clinical trials in the future. Clinical trials of FN - α5β1 integrin targeted drugs are described in detail in **Table 1**.

### HA-CD44

The binding of HA derived from CAMs to CD44 expressed on OC plays a significant part in promoting tumor metastasis and chemoresistance. Formerly, HA-based drugs have been shown to have anticancer activity in human OC nude mouse xenograft models (65). A study showed that CD44 targeting HA nanoparticles successfully delivered MDR1 siRNA into OC cells, and the nanoparticles combined with paclitaxel improved the sensitivity of MDR cells to paclitaxel and overcome the chemoresistance of OC (66). Subsequently, various HA-conjugated nanomedicines were developed to delivery chemotherapeutic agents such as Granzyme B, paclitaxel and FAK siRNA (67, 68). Some clinical trials have demonstrated the safety and tolerance of HA-based nanoconstructs in colon cancer (69). In the future, clinical trials are needed to further explore the efficacy of CD44 targeting HA-conjugated nanomedicines in the treatment of OC.

### HGF

HGF derived from OC promoted the premature senescence of normal mesothelial cells. Senescent mesothelial cells facilitated mesothelial clearance and tumor angiogenesis. A separate study showed HGF led to chemoresistance of OC by upregulating the MET/PI3K/Akt pathway (70). Rilotumumab (AMG 102) is an anti-HGF monoclonal antibody developed to neutralize the biological activity of HGF, thus blocking the HGF/MET pathway. However, in a phase II clinical trail, rilotumumab monotherapy showed limited benefit in patients suffering recurrent or persistent OC (71). This implies that HGF inhibitor combined with other therapeutic strategies may potentially improve efficacy and overcome chemoresistance.

## Other drugs mechanism effect CAMs for OC treatment

Several studies have shown that drugs normally used to treat tumors can also modulate CAMs, such as vitamin D, metformin, tamoxifen and so on.

### Vitamin D

Some epidemiological studies suggest that low circulating level of vitamin D is related to poor outcome in patients with various cancers (72, 73). In a meta-analysis of randomized controlled trials, vitamin D supplementation therapy can significantly reduce cancer-related mortality (74). A recent study found that vitamin D inhibited the EMT of mesothelial cells to suppress tumorigenesis in OC (75). Mechanistically, vitamin D inhibited thrombospondin-1 expression by suppressing Smad-dependent TGF-β signaling through VDR-SMad3 competition, which blocking the interaction between CAMs and cancer cells. In particular, the stabilized mesenchymal state of CAMs was restored to its normal epithelial state of preventing cancer cell adhesion and growth by adding vitamin D. Moreover, vitamin D was confirmed to reduce MMPs secretion in cancer-associated mesothelial cells. The inhibition of TGF-β signaling and MMPs secretion can enhance the efficacy of immune checkpoint inhibitors (76, 77). These results displayed that the combination of vitamin D and chemotherapy may be effective in advanced ovarian cancer.

### β-Escin

β-Escin is the main active component in horse chestnut seed extract, and its anticancer activity has also been reported in various cancers. A study using a three-dimensional quantitative high-throughput screening platform (3D-qHTS) to screen 2420 naturally extracted compounds found thatβ-escin can effectively suppress migration and viability of OC cells *in vitro*. In further mechanistical study, β-escin treatment regulated HIF1α stability and reduced the expression of fibronectin, laminin-C1, tenascin, and collagen1-a2 in CAMs in mouse, which contributing to the decreased ability of OC cells to adhere and invade (78).

### Metformin

Metformin is a common drug used to treat type 2 diabetes. Recent epidemiological studies have shown that metformin has antitumor effects. A prospective phase II clinical trial found that metformin treatment was well tolerated in nondiabetic OC patients and contributed to better median overall survival (OS) (79). Metformin may target multiple immune cells in OC, such as T cells, myeloid-derived suppressor cells (MDSCs), neutrophils and macrophage (80–83). Recently, metformin was reported to alter CAMs in the omental microenvironment (84). Metformin inhibited the expression of tricarboxylic acid (TCA) enzyme succinyl CoA ligase (SUCLG2), activated prolyl hydroxylases (PHDs), finally leading to the inhibition of TGF-β-driven metabolic upregulation of HIF1α in CAMs. The degradation of HIF1α contributed to reducing CCL2 and IL-8 production, thereby blocking the invasion of OC cells to mesothelial cells.

### Acacetin

Acacetin is a natural flavonoid widely found in vegetables. Previous studies suggested that acacetin showed anti-cancer efficacy in various cancers. In a mouse model of gastric cancer, acacetin treatment delayed the development of peritoneal metastasis *via* inhibiting PI3K/Akt/Snail signal pathway (85). Recently, emerging evidence has confirmed that acacetin inhibits CAMs-evoked malignant characteristics and reduces PCNA and MMPs secretion, which suppressing the proliferation and invasion of OC cells (86). Mechanically, acacetin can suppress LPA secretion in CAMs and further block the activation of receptor for advanced glycation end-products (RAGE)-PI3K/AKT signaling in OC cells. Moreover, acacetin decreased the secretion of pro-inflammatory cytokine IL-6 and IL-8 production in CAMs.

### L-carnosine

L-carnosine is a dipeptide widely distributed in human tissues, and it has anti-senescence and anti-cancer properties. Some studies showed that L-carnosine prolonged the replication life of somatic cells and inhibited the growth of cancer cells *in vitro* and *in vivo* (87). Interestingly, a previous report found that L-carnosine retarded senescence of human peritoneal mesothelial cells and suppressed progression of OC cells (88, 89). As mentioned earlier, mesothelial cells are peculiarlysusceptible to oxidative stress, which facilitates their senescence. L-carnosine can reduce mitochondrial oxidative stress by improving the cell’s ability to produce ATP, thereby leading to a compensatory reduction in mitochondrial biogenesis and superoxide production. Moreover, L-carnosine decreases various pro-cancerogenic factors secretion by CAMs, such as IL6, IL8, GRO1, PAI 1 and TGFβ1.

### HSVTK-modified CAMs

CAMs can be also used as drug carriers to enhance antitumor effects. A previous study engineered CAMs with the herpes simplex virus thymidine kinase/ganciclovir (HSVTK/GCV) system (90). Engineered CAMs can deliver the HSVTK bystander effect to human OC cells and induce the apoptosis of cancer cells. Intraperitoneal administration of HSVTK-expressing CAMs resulted in reduced tumor growth and prolonged survival in mouse model of OC. Moreover, distribution studies showed that engineered CAMs were preferentially located in tumor sites.

### Tamoxifen

Tamoxifen is an estrogen receptor modulator that has been shown to be used in the treatment of chronic peritoneal diseases. In mice peritoneal dialysis model, tamoxifen blocked TGF-β1-induced MMT of normal mesothelial cells, thereby inhibiting peritoneal fibrosis (91). Tamoxifen also inhibited GSK-3β/β-catenin signal pathway to attenuate peritoneal fibrosis (92). In the future, tamoxifen can be used as a prevention against mesothelial cells transformation in improving the treatment of OC peritoneal metastasis (93).

## Eficiencies and prospects

OC is a fatal disease with a high recurrence rate and a low 5-year survival. Immunotherapy has a lower successful ratio in OC compared with other immunogenic tumors, such as non-small cell lung cancer (NSCLC) and melanoma. In the last two decades, improvements in surgical approaches and the development of chemotherapeutic agents have led to improved survival rates in patients with advanced ovarian cancer. However, cytotoxic drug therapy is non-selective and usually results in transient antitumor responses and significant toxicity. The vast majority of women with ovarian cancer develop drug resistance after receiving first-line chemotherapy (94). There is increasing evidence that TME plays an important role in shaping tumor heterogeneity and drug resistance (95). Some studies have analyzed the feasibility of modifying TME as a treatment for OC. Exploring immunotherapies targeting the components of TME, such as dysfunctional immune cells, exosomes, CAMs and metabolites, would help to develop immunotherapies in OC. Mesothelial cells are the major components of OC microenvironment. They are arranged in the viscera and wall of peritoneal cavity, and are widely present in malignant ascites. Some studies have found that CAMs are closely related to the intraperitoneal metastasis, chemical resistance and tumor recurrence of OC (32, 33). In addition, some therapies that attempt to target CAMs have proved effective. For example, in a preclinical study, therapeutic targeting of CAMs-derived OPN enhances cisplatin response by increasing drug concentrations and DNA damage in OC cells (32). Blocking the interaction of CAMs with OC cells by using neutralizing antibody or aptamers has also been shown to be effective *in vivo* and *in vitro*. The review highlighted the key role of CAMs in the progression and prognosis of OC. We also described the progress of CAMs targeted therapy for OC. As the understanding of the mechanisms by which the TME effects OC progression and metastasis continue to improve, new therapeutic targets will be identified and validated, potentially contributing to the development of new and effective therapeutic regiments.

## Author contributions

AZ wrote original draft, drew the figure and made the summarizing table. YW, YZ and TZ corrected the draft and wrote the final version. All authors have read and agreed to the published version of the manuscript.

## Acknowledgments

We acknowledge the editors and the reviewers for insightful suggestions on this work.

## Conflict of interest

The authors declare that the research was conducted in the absence of any commercial or financial relationships that could be construed as a potential conflict of interest.

## Publisher’s note

All claims expressed in this article are solely those of the authors and do not necessarily represent those of their affiliated organizations, or those of the publisher, the editors and the reviewers. Any product that may be evaluated in this article, or claim that may be made by its manufacturer, is not guaranteed or endorsed by the publisher.
